# Development of a dosing algorithm integrating pharmacogenetic markers and inflammation for individualization of initial voriconazole maintenance doses

**DOI:** 10.1128/aac.00278-26

**Published:** 2026-09-11

**Authors:** Elodie Gautier-Veyret, Othilie Giannoni, Marc Manceau, Capucine Arrive, Sylvia Klomp, Matthieu Roustit, Fabien Lamoureux, Benjamin Hennart, Caroline Solas, Sarah Baklouti, Celine Verstuyft, Christelle Boglione-Kerrien, Jesse J. Swen, Nicolas Pallet, Anette Veringa, Jan Willem C. Alffenaar, Takahiro Yamada, Takafumi Naito, Françoise Stanke-Labesque, Charles Khouri, on behalf of the Therapeutic Drug Monitoring and Personalization Treatment group of the French Society of Pharmacology and Therapeutics and the French-Speaking Network of Pharmacogenetics (RNPGx)

**Affiliations:** 1 Univ. Grenoble Alpes, INSERM U1300, HP2 Laboratory, Grenoble, France; 2 Univ. Grenoble Alpes, CHU Grenoble Alpes, Laboratoire de Pharmacologie, Pharmacogénetique et Toxicologie 36724 , Grenoble, France; 3 Réseau Francophone de Pharmacogénétique (RNPGX), Hôpital Européen Georges-Pompidou (HEGP), Paris, France; 4 Groupe Suivi Thérapeutique Pharmacologique-Personnalisation des traitements (STP-PT) de la Société Française de Pharmacologie et Thérapeutique (SFPT), Tours, France; 5 Pharmacie des Hôpitaux Universitaire de Genève, Geneva, Switzerland; 6 Section des Sciences Pharmaceutiques, Institut des Sciences Pharmaceutiques de Suisse Occidentale, Université Genève Suisse 69610 , Geneva, Switzerland; 7 Univ. Grenoble Alpes, Inserm CIC1406, CHU Grenoble Alpes 36724 , Grenoble, France; 8 Department of Clinical Pharmacy and Toxicology, Leiden University Medical Center 4501, Leiden, Netherlands; 9 UNIROUEN, INSERM U1096, CHU Rouen, Department of Pharmacology, Normandie University, Rouen, France; 10 CHU Lille, Service de Toxicologie et Génopathies, Lille, France; 11 Laboratoire de Pharmacocinétique et Toxicologie, Hôpital La Timone, Aix-Marseille University, APHM, UMR "Emergence des Pathologies Virales" Inserm 1207 IRD 190 128791, Marseille, France; 12 Laboratoire de Pharmacocinétique et Toxicologie, IFB, Hôpital Purpan 54904, Toulouse, France; 13 Service de Génétique Moléculaire, Pharmacogénétique et Hormonologie de Bicêtre, Hôpitaux Universitaires Paris-Saclay, Assistance Publique-Hôpitaux de Paris 378965, Le Kremlin-Bicêtre, France; 14 Department of Clinical and Biological Pharmacology and Pharmacovigilance, Pharmacoepidemiology and Drug Information Centre, Rennes University Hospital 36684, Rennes, France; 15 Service de Biochimie, Assistance Publique Hôpitaux de Paris, Assistance Publique Hôpitaux de Paris 26930 , Paris, France; 16 Department of Clinical Pharmacy and Pharmacology, University Medical Center Groningen, University of Groningen 666253, Groningen, the Netherlands; 17 Department of Clinical Pharmacy, OLVG 10215 , Amsterdam, the Netherlands; 18 Department of Hospital Pharmacy, Hamamatsu University School of Medicine 12793, Hamamatsu, Japan; 19 Department of Pharmacy, Shinshu University Hospital 623097, Matsumoto, Nagano, Japan; 20 Univ. Grenoble Alpes, Pharmacovigilance Unit, Grenoble Alpes University Hospital 36724, Grenoble, France; University Children's Hospital Münster, Münster, Germany

**Keywords:** voriconazole, pharmacogenomics, inflammation, pharmacokinetics, model-informed precision dosing

## Abstract

Achieving early therapeutic trough concentrations (Cmin) of voriconazole (VRC) is crucial for treatment efficacy and to avoid side effects. Since VRC Cmin are influenced by numerous determinants, this multicenter retrospective study aimed to develop a multiparametric dosing algorithm for individualizing initial VRC maintenance doses. Individual data regarding VRC Cmin from adult patients and associated parameters (age, sex, underlying disease [presence of hematological disease/other], weight, dose, route of administration, indication: curative/prophylactic, cytochrome [CYP]2C19 and 3A genotypes, C-reactive protein [CRP], transaminases, bilirubin, and proton pump inhibitor treatment) were collected from six previously published studies and routine care data from seven laboratories (building cohort). A mixed-effects model predicting VRC Cmin was developed on this building cohort using a stepwise cross-validation procedure and then externally validated on an independent cohort (validation cohort). The building and validation cohorts included 977 VRC Cmin in 277 patients and 47 VRC Cmin in 33 patients, respectively. The initial model, which integrated VRC daily dose, CYP2C19 genotype, and CRP, was enriched by two additional covariates: age and underlying disease. The final model had an *R*² of 0.69 but tended to underestimate low VRC Cmin and overestimate high VRC Cmin. Finally, a formula derived from this final model was proposed and integrated into an Excel sheet to individualize initial VRC maintenance doses. The developed predictive model allowed for the creation of a multiparametric dosing algorithm for the individualization of initial VRC maintenance doses. The benefit of this algorithm will soon be evaluated in a prospective pilot study to improve initial VRC exposure.

## INTRODUCTION

Invasive aspergillosis is an invasive fungal infection occurring primarily in immunocompromised patients. The azole antifungal agent voriconazole (VRC) is the standard of care for its treatment and prevention (1, 2). Despite adequate management, invasive aspergillosis mortality remains extremely high, ranging from 19 to 61% in hematological patients (3). This high mortality is partially attributable to inadequate drug exposure (4). Indeed, VRC exhibits substantial pharmacokinetic variability, a well-established concentration-effect relationship, and a narrow therapeutic index (5, 6). Subtherapeutic concentrations increase the risk of treatment failure, while supratherapeutic concentrations elevate the risk of adverse effects and treatment discontinuation (6, 7). Consequently, American and European guidelines recommend therapeutic drug monitoring (TDM) to maintain VRC trough concentrations (VRC Cmin) within the therapeutic range (2, 8). However, VRC dose adjustment based on standard TDM can only occur after 3–5 days, which is the minimum time required to reach the pharmacokinetic steady state and measure VRC Cmin. Since invasive aspergillosis is a lethal infection, achieving therapeutic VRC Cmin immediately upon treatment initiation is critical, as advocated for other fungal infections (9–12).

The interpatient pharmacokinetic variability of VRC is explained in part by genetic polymorphisms of the cytochrome P450 (CYP) 2C19. Hence, genotype-guided dosing strategies have been proposed, successfully reducing the incidence of insufficient VRC Cmin (13, 14). However, these genotype-guided approaches do not fully account for all determinants of VRC Cmin. Multiple factors influence VRC exposure, including inflammatory status (15–17), genetic variants in *CYP3A* (18–20), age (21, 22), drug-drug interactions (with proton pump inhibitor [PPI] in particular) (23), and hepatic dysfunction (24). Specifically, numerous previous studies have demonstrated that the impact of the CYP2C19 genotype (± CYP3A genotype) on VRC exposure may be compromised by inflammation (25–27). Consistent with this, our prior prospective study demonstrated that pharmacogenetic markers alone were insufficient to predict initial VRC Cmin in curatively treated patients (27). This may be due to the frequent inflammatory status observed in patients with invasive aspergillosis (28, 29), with inflammation being identified as an independent risk factor of VRC overdose (30).

All these data therefore suggest that *a priori* dose adjustment strategies must integrate the patient’s inflammatory status in addition to the CYP2C19 genotype. This multimodal approach is particularly relevant in patients with invasive aspergillosis, where inflammation (known to induce a phenoconversion of VRC metabolism [16, 17, 31]) is commonly present (28, 29). Therefore, the objective of this study is to develop and validate a multiparametric dosing algorithm for the individualization of the first VRC maintenance doses, incorporating pharmacogenetic markers, inflammatory status, and any other relevant covariates.

## MATERIALS AND METHODS

### Study design

The cohort used to build the model (named “building cohort”) consisted of individual patient data from two main sources: cohort 1 corresponding to data from six published studies (16, 27, 30, 32, 33), which had been previously collected (15), and cohort 2 corresponding to routine care data from seven different laboratories affiliated with the French Society of Pharmacology and Therapeutics (*Société Française de Pharmacologie et Thérapeutique*) and/or the French Network of Pharmacogenetics (*Réseau Francophone de Pharmacogénétique*). Following internal validation, the established model was externally validated using an independent set of routine care data sourced from the Biobank Hematologische Ziekten at the Leiden University Medical Center (cohort 3 named “validation cohort”). The origin and use of cohorts 1, 2, and 3 are detailed in Supplemental material (Fig. A1).

The inclusion criteria for all data sources were adult patients treated with VRC (intravenous or oral administration) with a known CYP2C19 genotype ± CYP3A4 and CYP3A5 genotypes. Patients also required at least one pair of VRC Cmin (determined at the pharmacokinetic steady state) and C-reactive protein (CRP) level determined concomitantly (maximal delay of ±48 h between VRC Cmin and CRP measurements). Exclusion criteria were missing data for VRC Cmin, VRC dose, or CRP levels, aberrant VRC Cmin (>20 mg/L suggesting contamination), or presence of major drug-drug interactions (involving strong inhibitor/inducer). Regarding cohort 1, the studies were selected following a systematic review, and the complete study selection criteria were previously described (15). All contributors of cohort 1 were recontacted to retrospectively obtain additional data on VRC indication (prophylactic or curative treatment) and bilirubin levels. These two parameters, which can significantly influence VRC exposure (26, 27, 34), were not included in our previous study (15). Data for cohorts 2 and 3 were collected retrospectively from routine care.

### Clinical data collection

The following data were recorded: patient demographics (age, sex, and weight), clinical characteristics (underlying disease, specified as hematological malignancies or other), laboratory parameters (CRP level, aspartate aminotransferase, alanine aminotransferase, total bilirubin, and CYP2C19, 3A4, and 3A5 genotypes), VRC treatment details (date of VRC initiation, daily dose, route of administration, and concomitant PPI treatment), and pharmacokinetic data (VRC Cmin determined at pharmacokinetic steady-state, along with the date of blood collection). The analytical methods used for measuring CRP and VRC Cmin and determining CYP genotype were also noted. Data are indicated as median [minimal-maximal values] for quantitative data or numbers (percentages) for qualitative data. As cohorts 2 and 3 comprised retrospectively collected data from routine care, some data are lacking. The proportions of missing data are indicated in Tables 1 and 2.

**TABLE 1:** Patient characteristics in building and validation cohorts^*c*^

Characteristics^*a*^	Building cohort (*n* = 277)	Missing data (%)	Validation cohort (*n* = 33)	Missing data (%)
Age (years)	60.1 [18.8-85.5]	0	60.0 [19.0-74.0]	0
Male	180 (65)	0	20 (61)	0
Weight (kg)	65 [34-138]	4	77 [51-128]	9
Curative indication	178 (78.4)	18	na	100
Hematological malignancies	187 (83.5)	19	33 (100)	0
CYP2C19 phenotype^*b*^				
*Normal metabolizer*	116 (41.9)	0	8 (24.3)	0
*Intermediate metabolizer*	75 (27.1)		12 (36.4)	
*Poor metabolizer*	9 (3.2)		1 (3.0)	
*Rapid metabolizer*	70 (25.3)		11 (33.3)	
*Ultra-rapid metabolizer*	7 (2.5)		1 (3.0)	
CYP3A4 genotype				
*1/*22	26 (13.8)	32	na	100
CYP3A5 genotype				
*1/*1	10 (5.2)	31	na	100
*1/*3	25 (13.1)			
*3/*3	156 (81.7)			

^*a*^ Data are indicated as median [min-max] or numbers (%).

^*b*^ According to the CPIC guidelines (35).

^*c*^ CYP, cytochrome; na, not available.

**TABLE 2:** Description of the voriconazole trough plasma concentrations and associated parameters in building and validation cohorts^*b*^

Characteristics^*a*^	Building cohort (*n* = 977)	Missing data (%)	Validation cohort (*n* = 47)	Missing data (%)
VRC Cmin (mg/L)	1.7 [<0.1-11.2]	0	3.7 [0.2-116]	0
Daily dose (mg/day)	400 [52.0-1054]	0	400 [250-1200]	0
IV treatment	219 (23.1)	3.0	35 (74)	0
CRP (mg/L)	29 [0.01-436]	0	138.2 [1.2-512.3]	0
ASAT (UI/L)	28 [3.0-10360]	8.0	na	100
ALAT (UI/L)	31 [4.0-2605]	8.0	na	100
Total bilirubin (µmol/L)	8.0 [0.3-71.0]	25.0	na	100
Proton pump inhibitor cotreatment	666 (73)	7.0	40 (85)	0
Type of proton pump inhibitor				
*Pantoprazole*	245 (25.0)	30.0	na	100
*Omeprazole*	10 (0.01)			
*Esomeprazole*	164 (16.7)			
*Rabeprazole*	11 (0.01)			
*Lansoprazole*	7 (0.007)			

^*a*^ Data are indicated as median [min-max] or numbers (%).

^*b*^ ASAT, aspartate aminotransferase; ALAT, alanine aminotransferase; CRP, C reactive protein; IV, intravenous; na, not available; VRC Cmin, voriconazole plasma trough concentration.

### Laboratory analysis

Analytical methods used for VRC Cmin, CRP, and CYP genotyping utilized in each center/study are detailed in supplemental material (Table A1) . For VRC Cmin and CRP, values below the limit of quantification were imputed as the lower limit of quantification of the respective method. VRC Cmin was considered therapeutic between 1 and 5.5 mg/L (8). Regarding CYP genotyping, the following polymorphisms were analyzed: CYP2C19*2 (rs3758581), CYP2C19*3 (rs4986893), and CYP2C19*17 (rs12248560). In a subset of patients, CYP3A4*22 (rs35599367) and CYP3A5*1 (rs776746) were also genotyped. The CYP2C19 phenotype (poor [PM], intermediate [IM], normal [NM], rapid [RM], or ultra-rapid [UM] metabolizer) was determined from the CYP2C19 genotype for each patient according to the Clinical Pharmacogenetics Implementation Consortium (CPIC) recommendations (35). Subsequently, RM and UM patients were grouped to represent increased metabolic capacity, while IM and PM patients were grouped to represent decreased metabolic capacity. For CYP3A5, the CYP3A5*1 allele is associated with the expression of this CYP, whereas the CYP3A5*3 allele is associated with its non-expression.

### Model development and validation

A linear mixed-effects model predicting VRC Cmin was constructed from the building group. This model included a random intercept per patient to account for the multiple measures taken per individual and fixed effects for all other predictors. The model’s formula is presented below (Equation 1):


$$VRC\;Cmin_{i}=\sum_{j}\beta_{j}x_{ij}+\sum_{p}\nu_{p}\delta_{patient(i)}=p+\varepsilon_{i}$$

With *β*_*j*_ the coefficients of fixed effects (predictive variables); *ν*_*p*_ the random effects associated with patient *p*, with the *ν*_*p*_ distributed according to a centered Gaussian distribution with a common standard deviation; and *ε*_*i*_ the residual measurement error, distributed according to another centered Gaussian distribution with another standard deviation.

**Fig 1:**
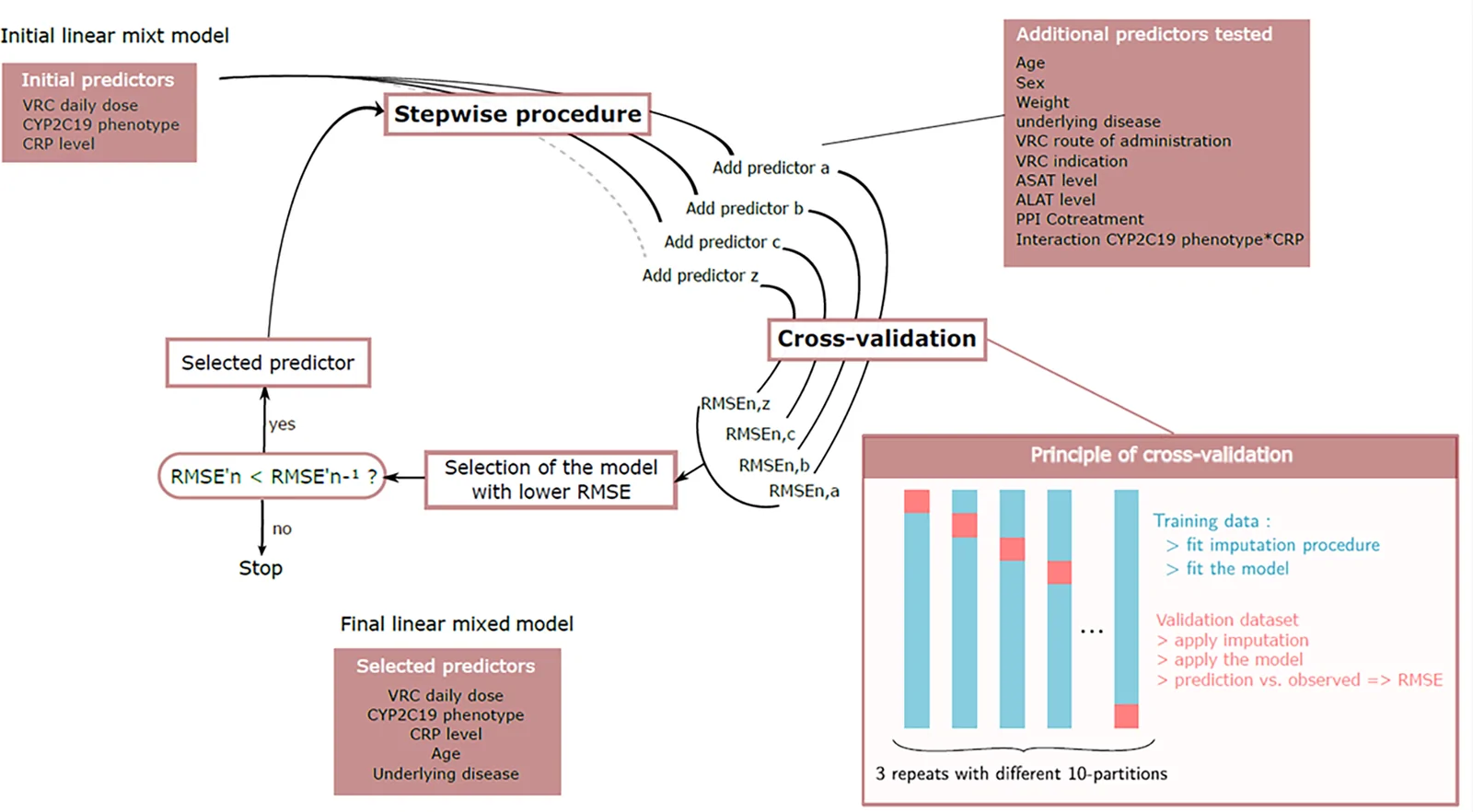
Schematic representation of the modeling development workflow. ASAT, aspartate aminotransferase; ALAT, alanine aminotransferase; CRP, C-reactive protein; CYP, cytochrome; PPI, proton pump inhibitor; VRC, voriconazole.

The workflow for model development is depicted in Fig. 1. Since this study aimed to individualize the VRC starting maintenance dose, the VRC daily dose was integrated as a covariate in the initial model. The CYP2C19 phenotype and CRP level were also incorporated, given their established roles as major determinants of VRC Cmin (26, 36). As the initial data set (cohort 1 and 2: building group) had missing data, an imputation strategy based on the K-nearest neighbors algorithm was employed, excluding variables with over 20% of missing data. Other predictors were selected based on a stepwise cross-validation procedure designed to minimize the residual mean squared error (RMSE) of the model, as illustrated in Fig. 1. Briefly, this procedure (repeated n times) consists of (i) adding to the initial model with initial residual mean standard error (RMSE_0_) one after one the additional predictors a, b, …, z; (ii) estimating the RMSE of secondary models (RMSE_n,a_; RMSE_n,b_; …; RMSE_n,z_) through a cross-validation; (iii) selecting among all generated secondary models those with the lower RMSE; and (iv) comparing the RMSE_n_ of the selected model to the RMSE_n-1_ of the previous model without the last added predictor. If RMSE_n_ is lower than RMSE_n-1_, the corresponding predictor is selected and the process of adding another predictor is repeated until the RMSE_n_ stops decreasing. The principle of cross-validation is illustrated in the box in the bottom right of the Fig. 1. After the imputation procedure and fitting the model on 9/10 of the data, predicted and observed VRC Cmin were compared on the remaining 1/10 of the data to estimate RMSE of each model. Such procedure was repeated three times, allowing estimation of mean RMSE and its standard deviation. In addition to conventional predictors (see list in Fig. 1), an interaction term between CRP and CYP2C19 phenotype was also assessed, based on previous works suggesting a differential effect of inflammation according to the CYP2C19 phenotype (37, 38). The software used to build the model was R v4.3.3.

Internal evaluation was conducted by comparing observed and predicted VRC Cmin, followed by an external evaluation using an independent data set (validation cohort = cohort 3). After both internal and external validations, the mathematical formula of the final model was extracted from R. The term for VRC daily dose was then isolated to derive the formula for the multiparametric algorithm. Detailed steps required to convert the formula of the final model into the formula to individualize VRC daily dose are detailed in the Supplemental file “Additional methodological considerations”. The resulting formula was integrated into a more user-friendly Excel calculator.

## RESULTS

### Characteristics of patients and voriconazole trough concentrations

Cohorts 1 and 2 used to build the linear-mixed-effects model predicting VRC Cmin included 277 patients for 977 VRC Cmin (building cohort), while the validation cohort consisted of 33 patients and 47 Cmin (validation cohort). Sources of these data are described in Supplemental material (Fig. A1). Study populations (building and validation groups) and VRC Cmin are respectively described in Tables 1 and 2. Most of the patients in the building cohort (78.4%) had hematological malignancies, and 83.5% were treated curatively with VRC, while all patients in the validation cohort received VRC after an allogeneic hematopoietic stem cell transplantation secondary to a hematological disease.

VRC Cmin ranged from 0.1 to 11.2 mg/L, with a median of 1.7 mg/L in the building cohort, while it varied from 0.2 to 11.6 mg/L, with a median of 3.7 mg/L in the validation cohort (Table 2). Moreover, 61.1% and 68% of VRC Cmin were in the therapeutic range in the building and validation cohorts, respectively.

### Model development

The stepwise procedure (detailed in Fig. 1) enriched our initial model (integrating VRC daily dose, CYP2C19 phenotype, and CRP level) by incorporating age and underlying disease as predictors. Further addition of variables to the model led to a modest decrease in the RMSE estimated based on our repeated 10-fold cross-validation (see Fig. A2 in Supplemental material), with additional predictors that varied. The term interaction “CRP*CYP2C19 genotype” was especially not retained. The RMSE of our initial model (1.75) was reduced to 1.71±0.03 (Fig. A2 in Supplemental material). Table 3 indicates estimates of each predictor retained in the final model. The final model’s *R*^2^ was 0.69, indicating that our model explained 69% of VRC Cmin variability.

**TABLE 3:** Estimates of predictors retained in the final linear mixed-effects model^*a*^

	Estimate	Standard error
Intercept	2.37	0.099
Log VRC daily dose	0.57	0.066
CYP2C19 phenotype_ RM/UM	−0.43	0.11
CYP2C19 phenotype_IM/PM	0.083	0.10
CRP	0.98	0.053
Age_first-order polynomial	0.60	0.094
Underlying disease_other	0.23	0.10
Age_second-order polynomial	0.31	0.090

^*a*^ CRP, C reactive protein; CYP, cytochrome; IM, intermediate metabolizer; PM, poor metabolizer; UM, ultra-rapid metabolizer; VRC, voriconazole.

### Model evaluation

**Fig 2:**
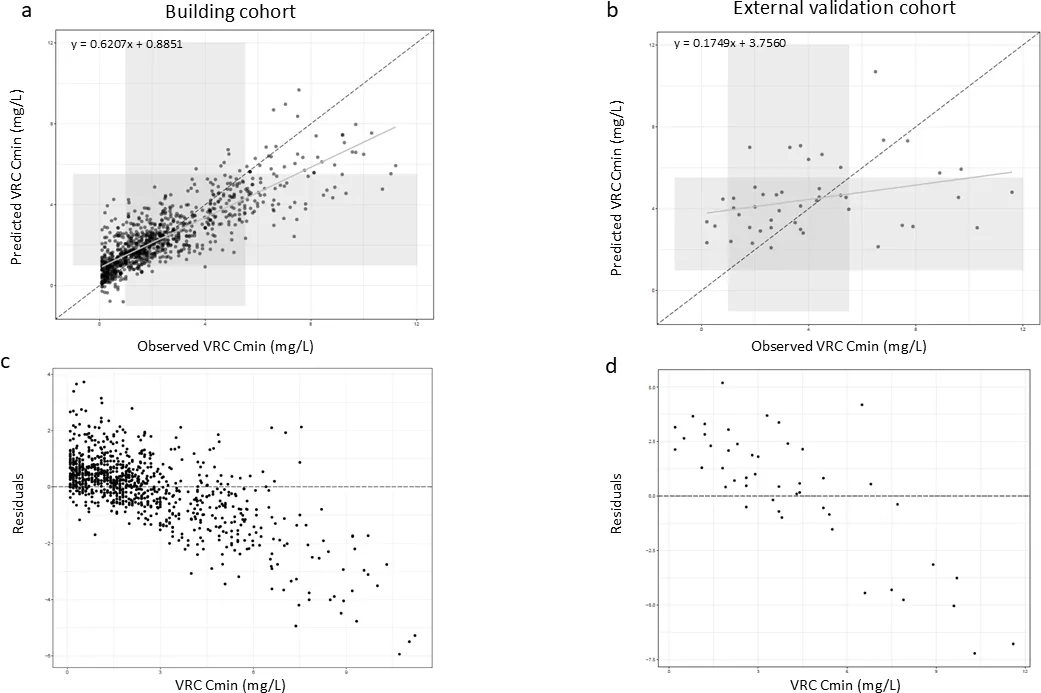
Goodness-of-fit plots of the final model. Observed versus predicted voriconazole trough concentrations (VRC Cmin) in building (**a**) and external validation (**b**) cohorts. Residuals of the model versus VRC Cmin for the building cohort (**c**) and external validation cohort (**d**). Gray zones and solid gray lines indicate the therapeutic range of VRC Cmin and trend lines for panels a and b.

Goodness-of-fit plots of the final model applied to building and validation cohorts are presented in Fig. 2. The individual predicted VRC Cmin appears to be distributed around the line of unity for the building cohort (Fig. 2a), but not for the external validation cohort (Fig. 2b). Nevertheless, predicted VRC Cmin seems slightly underestimated at subtherapeutic concentrations and overestimated at supratherapeutic concentrations for both building and external validation cohorts. This bias is confirmed by the residuals analysis (Fig. 2c and d). Specifically, the residuals are influenced by VRC Cmin, displaying positive residuals for subtherapeutic VRC Cmin and negative VRC Cmin for supratherapeutic VRC Cmin. Considering a therapeutic window of 1–5.5 mg/L for VRC Cmin, 75.8% and 63.8 % of VRC Cmin in the respective building and external validation cohorts were well categorized by the model (in or out of this target). Most of the model’s prediction errors concerned VRC Cmin predicted within the therapeutic interval, whereas the observed VRC Cmin were not (21.5% and 23.4 % of VRC Cmin in the respective building and external validation cohorts).

### Algorithm for starting dose model

After exclusion of the term “patient” and isolating “daily dose” from Equation 1, the formula of the final model yields the following formula (Equation 2):


$$\begin{array}{rl} \text{Daily dose}=e^{\wedge }( & 0.6028\times VRC\;C_{min}-0.007924\times [CRP]-0.0007523\times {\text{Age}}^{\wedge }2+0.05011\times \text{Age}\;+\\ & 4.298+[(-0.1176\text{ for }CYP2C19\;IM/PM)\text{ or }(0.5456\text{ for }CYP2C19\;UM/URM)\text{ or }(0\text{ for }CYP2C19\;EM)]\;+\\ & [0.3657\text{ for haematological patients or }0\text{ for other patients}]) \end{array}$$

with daily dose expressed in mg/day, VRC Cmin and CRP expressed in mg/L, age in years. VRC Cmin is the VRC Cmin targeted that must be between 1 and 5.5 mg/L. The different steps linking the final model to Equation 2 are detailed in supplementary material (see Suppl. file “Additional methodological considerations”).

Finally, Equation 2 was integrated into an Excel calculator (VORI_CALCULATOR in Additional results), designed for straightforward use by clinicians. Users must input the patient’s date of birth (allowing age calculation), his/her CYP2C19 phenotype (deduced from the CYP2C19 genotype), the CRP level determined at treatment initiation, and the underlying disease (hematological malignancies/other) to obtain the individualized VRC maintenance daily dose. The default target VRC Cmin is 2.5 mg/L, but clinicians can adjust. Since VRC is administered twice daily, the individualized daily dose is divided by 2 to determine the unit dose administered every 12 h. To further facilitate implementation, the Excel VORI_CALCULATOR also specifies the volume of oral suspension for each dose or the nearest rounding dose using the available tablets (50 and 200 mg per tablet).

## DISCUSSION

Managing VRC treatment presents significant challenges due to its high inter- and intra-patient pharmacokinetic variability driven by numerous determinants, a narrow therapeutic range, and the risk of severe side effects. Consequently, VRC exposure frequently falls outside the therapeutic range during the initial days of therapy (13, 14, 18, 39), potentially exposing patients to treatment failure or adverse effects. Therefore, *a priori* dose adjustment strategies could be a highly valuable tool for improving VRC efficacy and safety. While several previously described strategies of VRC initial dose individualization relied solely on CYP2C19 genotype or inflammatory status (25), we developed and internally and externally validated a multiparametric dosing algorithm. The established formula in our algorithm incorporates the CYP2C19 phenotype (deduced from genotype) and CRP level, which appear to be the two primary determinants of VRC exposure (26, 36), but also the patient’s age and underlying disease (hematological patients or other).

The numerous prior studies reporting the impact of CYP2C19 genotype on VRC exposure (33, 40) led the CPIC to establish therapeutic recommendations for VRC dose adjustment based on CYP2C19 genotype (35). More recently, several initiatives reported successful implementation of CYP2C19 genotyping for initial VRC dose adjustment in prophylaxis (13, 14, 41), with a significant reduction in insufficient VRC Cmin (13, 14). However, despite the CYP2C19-guided dosing, VRC Cmin outside the therapeutic range was still not uncommon in these studies. Indeed, 29% to 36% of patients included in the studies conducted at the Levine Cancer Institute had VRC Cmin < 1 mg/L (with this proportion reaching approximately 50% in CYP2C19 NM) (13, 42), while 14% (6/44) of CYP2C19 RM receiving increased VRC dose in the Hicks study had VRC Cmin higher than 5.5 mg/L (14). Such findings strongly suggest that integrating additional determinants of VRC exposure could further refine *a priori* VRC dose adjustment strategies.

Here, we have *a priori* integrated the CRP level (determined concomitantly with VRC Cmin measurement) into the model. This was based on the extensive literature describing the negative effect of inflammation on CYP2C19 activity (17, 43), which leads to increased VRC exposure (16, 30), irrespective of the CYP2C19 genotype (36, 37). As inflammation is an independent risk factor for VRC overdose (30, 44) and side effects (44), incorporating inflammatory status into *a priori* VRC dose adjustment approaches could theoretically reduce the incidence of supratherapeutic VRC Cmin and treatment discontinuation. Furthermore, several studies have reported differential effects of inflammation according to the CYP2C19 genotype (37, 38, 45). To investigate this hypothetical interaction between CYP2C19 genotype and inflammatory status, an interaction term “CRP*CYP2C19 genotype” was tested. This term was ultimately not incorporated into the final model due to a modest decrease in the RMSE. This might seem contradictory to previous works reporting a differential effect of inflammation according to the genotype-predicted CYP2C19 phenotype (37, 38, 45). However, this may simply reflect that the interaction between inflammation and basal CYP2C19 phenotype is not being clinically relevant enough, as already suggested by Klomp et al. (45). Further research is likely needed to elucidate this question.

Beyond CYP2C19 genotype and CRP level, the final model also incorporated patient age and underlying disease. The positive influence of age on VRC exposure in adult patients is already well described (21, 22, 42), corresponding to reduced hepatic clearance (46) and CYP2C19 activity (47) in older patients. The selection of the covariable “age” in the final model is thus logical and consistent with a previous study establishing a predictive model for VRC maintenance dose (36). Regarding the impact of the underlying disease (hematological patients vs. others), it is plausible that the patient’s condition influences VRC pharmacokinetics. According to our multiparametric algorithm, patients with hematological malignancies required higher VRC doses than patients with other immunocompromised diseases. This finding aligns with a retrospective study reporting different VRC exposure according to clinical departments (48) and could be related to pharmacokinetic alterations specific to hematological patients (e.g., decreased bioavailability due to mucositis and/or drug-drug interaction with letermovir or chemotherapy).

In the final model, sex, bilirubin, CYP3A4 and CYP3A5 genotype, weight, and concomitant use of PPIs were not retained, contrary to previous studies that had demonstrated a link between VRC exposure and these covariables (21–23, 36). This may be partly explained by missing data, especially for bilirubin and CYP3A genotype. For PPIs, the exact PPI was known in only 45% of the cases. The combination of these lacking data and the PPI pantoprazole not being a CYP2C19 inhibitor (49) may explain why PPI cotreatment was not selected in the final model, as is consistent with another study (36).

Overall, our model accounted for 69% of the VRC Cmin variability, a reasonable result that supports its future clinical application. However, future use of our algorithm should be reserved for patients with characteristics similar to the population used to develop the model, especially in terms of age and CRP (see ranges in Tables 1 and 2). Furthermore, the *R*² of our model is lower than that of a previously described model (50), and both internal and external evaluations showed a consistent underestimation at subtherapeutic VRC Cmin and overestimation at supratherapeutic levels, with prediction errors relatively frequent. These findings, suggesting that key sources of variability are not adequately captured, highlight the significant challenge in accurately predicting VRC exposure (51–53). Given these modest predictive performances, TDM remains essential within 3 to 5 days after applying our personalized dosing algorithm. Such verification is all the more essential as inflammation could vary very quickly in the early course of invasive aspergillosis.

Our study had several strengths. First, we utilized a large data set from multiple different centers to build our model, with most data obtained from routine care. Second, our model benefited from both internal and external validation. While such validation is essential for thorough evaluation of model-informed precision dosing (54), it was rarely performed or reported (36).

The current study has limitations. The available data sets used to build and validate our model contained only VRC Cmin, which is not the ideal marker of global exposure (i.e., area under the curve). However, VRC Cmin, which is the marker of VRC exposure used in routine care, was highly correlated with the area under the curve of VRC (55), mitigating this limitation. Secondly, the linear mixed model developed here differs from usual pharmacokinetic models and did not allow estimation of individual pharmacokinetic parameters, especially the volume of distribution. Hence, only VRC starting maintenance doses could be individualized with our algorithm, not loading doses. As the pharmacokinetic variability of VRC is mainly related to variation in its clearance, individualization of maintenance dose is probably sufficient to optimize VRC therapy. Thirdly, some variables previously described as predictors of VRC Cmin (bilirubin and albumin levels, CYP3A genotype, or some co-medications like PPI or letermovir) were lacking in available data sets and thus could not be integrated into our model. Letermovir, which is now widely used in recipients of allograft hematopoietic stem cell transplants, was especially associated with reduced VRC exposure (42, 56). Hence, the utility and safety of our algorithm should be evaluated in patients concomitantly treated with VRC and letermovir.

In conclusion, our study enabled the development and external validation of a multiparametric model for the individualization of the first maintenance doses of VRC. We integrated our multiparametric algorithm into an easily usable Excel calculator (VORI_CALCULATOR in Additional results) for simple deployment in clinical units. The utility of this multiparametric algorithm is currently being assessed in an interventional pilot study conducted in patients with hematological malignancies (VORIPRECI Study: NCT06582186).

## Data Availability

The data that support the findings of this study are available from the corresponding author upon reasonable request.

## SUPPLEMENTAL MATERIAL

Supplemental material aac.00278-26-s0001.docx Fig. A1 and A2; Table A1.

Supplemental file aac.00278-26-s0002.pdf Additional methodological considerations.

Additional results aac.00278-26-s0003.xls VORI_CALCULATOR Excel sheet integrating the multiparametric algorithm.

ASM does not own the copyrights to Supplemental Material that may be linked to, or accessed through, an article. The authors have granted ASM a non-exclusive, world-wide license to publish the Supplemental Material files. Please contact the corresponding author directly for reuse.
