# Supplementary material for: Development of a dosing algorithm integrating pharmacogenetic markers and inflammation for individualization of initial voriconazole maintenance doses
Source: Antimicrob Agents Chemother. 2026 Sep 11;70(10):e00278-26. doi: 10.1128/aac.00278-26 (PMC13689213; doi:10.1128/aac.00278-26)
Supplement: Supplementary file 1 [file aac.00278-26-s0002.pdf]

VORIPRED Study

Elodie Gautier-Veyret et al.

2026-05

Contents

1 Final model **2**

2 Formula **3**

2.1 Principle 3

2.2 Pre-processing : Polynomials of the age 3

2.3 Normalization of variables 4

2.4 Reversing the linear formula 4

The objective of this document is to provide a clear and reproducible way of getting the prediction formula derived in the VORIPRED project.

1 Final model

We decided to keep the following 7 predictors in the final version of the model, which we fit on the complete dataset.

```
## Linear mixed model fit by REML ['lmerMod']
## Formula: vrc ~ (1 | patient) + log_vrc_daily_dose + phenotype_URM.RM +
##     phenotype_IM.PM + crp_poly_1 + age_poly_1 + underlying_disease_other +
##     age_poly_2
##    Data: d_preprocessed
##
## REML criterion at convergence: 3632.7
##
## Scaled residuals:
##     Min      1Q  Median      3Q     Max
## -2.8829 -0.4843 -0.1024  0.3850  4.5975
##
## Random effects:
##  Groups   Name        Variance Std.Dev.
##  patient  (Intercept) 1.714    1.309
##  Residual             1.668    1.291
## Number of obs: 977, groups:  patient, 277
##
## Fixed effects:
##                          Estimate Std. Error t value
## (Intercept)               2.37008    0.09948  23.825
## log_vrc_daily_dose        0.56549    0.06560   8.620
## phenotype_URM.RM         -0.42764    0.11113  -3.848
## phenotype_IM.PM           0.08279    0.10077   0.822
## crp_poly_1                0.98238    0.05303  18.524
## age_poly_1                0.59853    0.09432   6.346
## underlying_disease_other  0.22571    0.09993   2.259
## age_poly_2                0.30923    0.08962   3.450
##
## Correlation of Fixed Effects:
##             (Intr) lg_v__ p_URM. p_IM.P crp__1 ag_p_1 undr__
## lg_vrc_dly_ -0.002
## phnt_URM.RM  0.041 -0.076
## phnty_IM.PM -0.067  0.042  0.391
## crp_poly_1  -0.023 -0.016  0.012  0.024
## age_poly_1  -0.088  0.080  0.015 -0.026 -0.025
## undrlyng_d_  0.054 -0.005  0.035 -0.054  0.087  0.030
## age_poly_2  -0.131  0.025  0.051 -0.093  0.027 -0.146 -0.139
```

2 Formula

2.1 Principle

We predict `vrc` using new values of explanatory variables, by applying:

1. the imputation step on missing data,
2. the transformation of variables (polynomials + normalization),
3. and then the model parameter fit on our dataset.

On the opposite, if we want to get the `vrc_daily_dose` allowing to obtain a given target `vrc` value, we need:

1. to apply the imputation step on missing data,
2. to transform variables (polynomials + normalization),
3. to reverse the linear regression formula,
4. this allows us getting a normalized value of `log_vrc_daily_dose`,
5. which we can transform (de-normalize) before applying the exponential function.

2.2 Pre-processing : Polynomials of the age

The polynomials of the age are built using the following code snippet, allowing us to compute the necessary coefficients to get `poly_1` and `poly_2`, the degree-1 and degree-2 polynomials that are used by the internal machinery of R.

```
## return a model matrix for data `x`
my_poly <- function (x) {
    degree <- 2
    ## check feasibility
    if (length(unique(x)) < degree)
        stop("insufficient unique data points for specified degree!")

    ## centring covariates (so that `x` is orthogonal to intercept)
    centre <- mean(x)
    x <- x - centre

    ## QR factorization of design matrix of ordinary polynomial
    QR <- qr(outer(x, 0:degree, "^"))
    ## X <- qr.Q(QR) * rep(diag(QR$qr), each = length(x))
    ## i.e., column rescaling of Q factor by `diag(R)`
    ## also drop the intercept
    X <- qr.qy(QR, diag(diag(QR$qr), length(x), degree + 1))[, -1, drop = FALSE]
    ## now columns of `X` are orthorgonal to each other
    ## i.e., `crossprod(X)` is diagonal
    X2 <- X * X
    norm2 <- colSums(X * X)    ## squared L2 norm
    alpha <- drop(crossprod(X2, x)) / norm2
    beta <- norm2 / (c(length(x), norm2[-degree]))
    colnames(X) <- 1:degree

    ## column rescaling so that `crossprod(X)` is an identity matrix
    scale <- sqrt(norm2)
    X <- X * rep(1 / scale, each = length(x))
    ## add attributes and return
    attr(X, "coefs") <- list(
        centre = centre,
        scale = scale,
        alpha = alpha[-degree],
```

```
        beta = beta[-degree]
    )
    X
}
my_poly_coefs <- attr(my_poly(d$age), "coefs")
```

In the excel file, we will need in particular the following values to compute the polynomials:

```
my_poly_coefs$centre
```

```
## [1] 53.01633
```

```
my_poly_coefs$scale
```

```
## [1]  485.7055 7740.8749
```

```
my_poly_coefs$alpha
```

```
## [1] -8.580456
```

```
my_poly_coefs$beta
```

```
## [1] 241.4635
```

2.3 Normalization of variables

We transform all other variables, by using our dataset knowledge to normalize their values:

In the excel file, we will need to center variables by subtracting the means:

```
##   phenotype_URM.RM phenotype_IM.PM crp_poly_1    age_poly_1    age_poly_2
## 1        0.3357216       0.2354145   61.01871 -2.562607e-18 -2.033919e-19
##   underlying_disease_other
## 1                0.1656943
```

We will also need to reduce the variables by dividing by the standard deviations:

```
##   phenotype_URM.RM phenotype_IM.PM crp_poly_1 age_poly_1 age_poly_2
## 1         0.472484       0.4244749   74.73205 0.03200922 0.03200922
##   underlying_disease_other
## 1                0.3720231
```

2.4 Reversing the linear formula

The next step consists in reversing and applying our prediction formula:

$$\text{vrc} = a_{\text{log\_vrc\_daily\_dose}} \times \text{log\_vrc\_daily\_dose} + \sum_i a_i X_i$$

Leading to:

$$\text{log\_vrc\_daily\_dose} = \frac{1}{a_{\text{log\_vrc\_daily\_dose}}} \times \text{vrc\_target} - \sum_i \frac{a_i}{a_{\text{log\_vrc\_daily\_dose}}} X_i$$

and finally:

$$\text{vrc\_daily\_dose} = \exp\left(\frac{1}{a_{\text{log\_vrc\_daily\_dose}}} \times \text{vrc\_target} - \sum_i \frac{a_i}{a_{\text{log\_vrc\_daily\_dose}}} X_i\right)$$

The excel file must contain the following values. The weight given to each variable is provided in front of each variable name. The weighted sum of all these values allows getting the `log_vrc_daily_dose` reduced/centered.

	x
(Intercept)	-4.1912248
phenotype_URM.RM	0.7562294
phenotype_IM.PM	-0.1464087
crp_poly_1	-1.7372340
age_poly_1	-1.0584378
underlying_disease_other	-0.3991441
age_poly_2	-0.5468359
vrc_target	1.7683859

Warning, in order to get the "true"(de-normalized) `log_vrc_daily_dose`, one must first multiply by the SD of `log_vrc_daily_dose` in our dataset: 0.3409, before adding the mean of the same variable in our dataset: 6.0645.

Finally, we apply the exponential function to get the `vrc_daily_dose` value.
