# Supplementary material for: Development of a dosing algorithm integrating pharmacogenetic markers and inflammation for individualization of initial voriconazole maintenance doses
Source: Antimicrob Agents Chemother. 2026 Sep 11;70(10):e00278-26. doi: 10.1128/aac.00278-26 (PMC13689213; doi:10.1128/aac.00278-26)
Supplement: Supplementary file 2 [file aac.00278-26-s0001.docx]

**Supplemental data**

Figure A1: Sources of data used to build and evaluate the model.

CRP, C reactive protein; Cmin, trough plasma concentration

Supplemental Figure A2. Evolution of the Residual Mean Squarred Error (RMSE) of the linear mixt model according to the numbers of predictors included in the model

The number of predictors indicated here are higher than the number of variables included in the model (which is 6 for VRC dose, CYP2C19 phenotype deducted from genotype, CRP level, age, underlying disease). First, categorical variables (e.g. the CYP2C19 phenotype) require including (n-1) dummy predictors, where n is the number of categories in the variable. Second, one numerical variable namely ‘age’ appears twice, with polynomials of first and second order included in the best model.

Supplemental Table A1: Analytical methods used for determination of biological parameters selected in the final model

|  | Gautier-Veyret *et al.*, 2015, 2019, 2020 ^2-4^ | Lamoureux *et al.*^5^ | Yamada *et al.^6^* | Veringa *et al.*^7^ | Center 1 | Center 2 | Center 3 | Center 4 | Center 5 | Center 6 | Center 7 | Cohort 3 (validation cohort from Klomp *et al.*^8^) |
| --- | --- | --- | --- | --- | --- | --- | --- | --- | --- | --- | --- | --- |
| VRC Cmin | LC-MS/MS | LC-MS/MS | HPLC-UV | LC-MS/MS | LC-MS/MS | HPLC-UV | HPLC-DAD | LC-MS/MS | LC-MS/MS | HPLC-UV | LC-MS/MS | LC-MS/MS |
| CRP | Immunoturbidimetry | Immunoturbidimetry | nephelometry | Immunoturbidimetry | Immunoturbidimetry | Immunoturbidimetry | Immunoturbidimetry | Immunoturbidimetry | Immunoturbidimetry | Immunoturbidimetry | Immunoturbidimetry | Immunoturbidimetry |
| Genotyping | Discrimination allelic | Discrimination allelic | PCR-RFLP | Discrimination allelic | Discrimination allelic | Discrimination allelic | Discrimination allelic | Discrimination allelic | Discrimination allelic | Sequencing (NGS) | Discrimination allelic | Discrimination allelic |
| CRP: C reactive protein; HPLC-DAD: High Performance Liquid Chromatography-diode array detection; HPLC-UV: High Performance Liquid Chromatography-Ultra Violet; LC-MS/MS : Liquid chromatography-tandem mass spectrometry; NGS : next generation sequencing; PCR-RFLP: Polymerase chain reaction - [Restriction Fragment Length Polymorphism; VRC Cmin: voriconazole trough concentration.](https://tice.agrocampus-ouest.fr/mod/glossary/showentry.php?eid=427) | | | | | | | | | | | | |

**References**

1. Bolcato L, Khouri C, Veringa A, *et al.* Combined Impact of Inflammation and Pharmacogenomic Variants on Voriconazole Trough Concentrations: A Meta-Analysis of Individual Data. Journal of Clinical Medicine 2021; **10**(10):2089.

2. Gautier-Veyret E, Fonrose X, Tonini J, *et al.* Variability of voriconazole plasma concentrations after allogeneic hematopoietic stem cell transplantation: impact of cytochrome p450 polymorphisms and comedications on initial and subsequent trough levels. *Antimicrob Agents Chemother* 2015; **59**: 2305–14.

3. Gautier-Veyret E, Truffot A, Bailly S, *et al.* Inflammation is a potential risk factor of voriconazole overdose in hematological patients. *Fundam Clin Pharmacol* 2019; **33**: 232–8.

4. Gautier-Veyret E, Thiebaut-Bertrand A, Roustit M, *et al.* Optimization of voriconazole therapy for treatment of invasive aspergillosis: Pharmacogenomics and inflammatory status need to be evaluated. *Br J Clin Pharmacol* 2021; **87**: 2534–41.

5. Lamoureux F, Duflot T, Woillard J-B, *et al.* Impact of CYP2C19 genetic polymorphisms on voriconazole dosing and exposure in adult patients with invasive fungal infections. *Int J Antimicrob Agents* 2016; **47**: 124–31.

6. Naito T, Yamada T, Mino Y, Kawakami J. Impact of inflammation and concomitant glucocorticoid administration on plasma concentration of triazole antifungals in immunocompromised patients. *Clin Chim Acta* 2015; **441**: 127–32.

7. Veringa A, ter Avest M, Span LFR, *et al.* Voriconazole metabolism is influenced by severe inflammation: a prospective study. *J Antimicrob Chemother* 2017; **72**: 261–7.

8. Klomp S, Manson M, Guchelaar, HJ, *et al.* Inflammation-Driven Variability in Drug Metabolism: Insights from Voriconazole treatment of HSCT Recipients. *Br J Clin Pharmacol* 2026 (*under press).*
